# Localization of Non-Linearly Modeled Autonomous Mobile Robots Using Out-of-Sequence Measurements

**DOI:** 10.3390/s120302487

**Published:** 2012-02-23

**Authors:** Eva Besada-Portas, Jose A. Lopez-Orozco, Pablo Lanillos, Jesus M. de la Cruz

**Affiliations:** Dpto Arquitectura de Computadores y Automatica, Universidad Complutense de Madrid, Av. Complutense s/n, 28040 Madrid, Spain; E-Mails: jalo@dacya.ucm.es (J.A.L.O.); planillos@fis.ucm.es (P.L.); jmcruz@fis.ucm.es (J.M.C.)

**Keywords:** autonomous mobile robots, location estimation, out-of-sequence, extended Kalman filter

## Abstract

This paper presents a state of the art of the estimation algorithms dealing with Out-of-Sequence (OOS) measurements for non-linearly modeled systems. The state of the art includes a critical analysis of the algorithm properties that takes into account the applicability of these techniques to autonomous mobile robot navigation based on the fusion of the measurements provided, delayed and OOS, by multiple sensors. Besides, it shows a representative example of the use of one of the most computationally efficient approaches in the localization module of the control software of a real robot (which has non-linear dynamics, and linear and non-linear sensors) and compares its performance against other approaches. The simulated results obtained with the selected OOS algorithm shows the computational requirements that each sensor of the robot imposes to it. The real experiments show how the inclusion of the selected OOS algorithm in the control software lets the robot successfully navigate in spite of receiving many OOS measurements. Finally, the comparison highlights that not only is the selected OOS algorithm among the best performing ones of the comparison, but it also has the lowest computational and memory cost.

## 1. Introduction

Autonomous mobile robots require to be localized, either in a local or global frame, in order to successfully perform different navigation tasks. With that purpose, they are usually equipped with (1) multiple sensors that provide redundant or complementary information about the robot location and (2) a localization module that is responsible for estimating on-line the robot location fusing the information provided by the sensors. The calculated location estimates are used in other modules of the robot control software that are in charge of deciding how the robot should act next. This makes the control signals applied to the robot actuators highly dependent on the location estimates. Therefore, it is extremely important to estimate the robot location correctly and efficiently.

**Figure 1.**
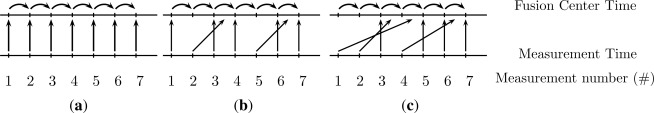
Out-Of-Sequence Problem. (**a**) Non-delayed data; (**b**) 1-step lag delay data; (**c**) N-step lag delay data.

In order to achieve both objectives, the location module can implement any of the sequential estimators that can deal with the uncertainty and characteristics associated to the robot dynamics and sensors, such as the Kalman, Information or Particle Filter (KF, IF, PF [1–4]). When we apply their basic formulations to the robot estimation problem, they sequentially estimate the current time robot location based on the current time measurements and previous time location estimate. Therefore, when the localization module implements the basic formulation of these filters (whose behavior is schematized in Figure 1(a), representing the dependency of the location estimate on the current time measurements with the arrows and on the previous time location estimate with the arcs), the location module requires to have all the measurements associated to the current time step before obtaining the estimate related to it.

However, the measurements often arrive delayed to the localization module, due to multiple factors such as the physical distribution of the sensors in some robotic problems, the communication network used to send information from the sensing modules to the localization one, and/or the time used to pre-process the raw measurements and extract the useful information that is sent to the localization module. The most difficult scenario appears when the delays and the sequence of the arrival of information to the localization module are not fixed, constituting the Out-Of-Sequence Problem (OOSP) [5]. The two main types of OOSP that can affect the sequential estimator of the localization module are presented in Figures 1(b) and 1(c). The former is the delayed *1-step lag* problem where the time stamp of the delayed measurements (#2 and #5) falls between the time stamps of the two previously arrived measurements (#1 and #3, and #4 and #6). The later is the general *OOS n-step lag* problem, where the measurements arrive delayed and OOS without any type of restrictions (#1 causes a 3-step lag OOSP, #2 causes a 1-step lag OOSP, and #4 causes a 2-step lag OOSP).

In order to deal with the measurement arrival delays, the localization module can basically implement four different solutions. The first, which consists on discarding the delayed measurements, is the easiest solution because it only requires to check the time stamp of the measurements. However, it is only useful for systems with spurious delayed measurements, because rejecting measurements increases the uncertainty of the robot location, affecting the stability and reducing the reliability of the robot control system [6–9]. The second approach, based on postponing the estimation until all the measurements are available, requires some extra memory to buffer the already received measurements and some knowledge about the sensors sampling rates and/or measurements maximum delays to decide when the estimation can be carried out [10]. However, it is only valid when the delays are small as it happens in [11], because the robot control system usually obtains the actions based on the current pose estimation and cannot wait to receive an estimate that is significantly delayed. The third approach consists in storing for all time instances the location estimates and measurements, rolling back to the time step associated with the time stamp of the measurement that has just arrived, and restarting the estimation process from that point. Therefore, it is the brute-force solution, used in [12–14], that lets the localization module obtain the same estimates as if it had received all the data without delays, at the expenses of increasing significantly the computational and memory requirements of the estimation algorithm. Finally, there is a fourth solution that avoids the computational burden of the brute-force approach and that is implemented in the localization module of the non-linearly modeled autonomous robot presented in this article. It consists in using (or developing) an OOS version of the filter that would have been used if there were no delays.

The estimation community has developed many OOS algorithms during the last decade in order to fulfill the requirements of an increasing number of sensor networks and tracking networked systems. Among the big number of possibilities, the choice of OOS algorithm to implement in the localization module of the robot depends on (1) the types of dynamic and sensorial models associated to the robot, and on (2) the nature of the out of sequence problems that the localization module faces. For instance, those autonomous robots whose dynamic and sensorial behaviors are modeled as linear systems with additive Gaussian noise can implement any of the big quantity of OOS Kalman or Information Filter variants (see [15] for a comprehensive review and comparison of centralized OOS KFs and OOS IFs, and [16–21] for decentralized OOS KFs). The smaller number of OOS variants for non-linearly modeled systems and the assumptions made during their development reduces the possibilities that exist for non-linearly modeled autonomous mobile robots. This work analyses the different possibilities, studies the performance of one of the most generic and computationally efficient existing approaches (which is adapted to take into account some peculiarities of a non-linearly modeled autonomous robot localization problem) and compares the selected approach against others.

This paper is organized as follows. Section 2 presents the analysis of the characteristics of the existing OOS filters for non-linear systems and studies the tracking non-linear modeled mobile object problems that have been tested with the analyzed OOS filters and that are close to the problem of estimating the location of an autonomous mobile robot with controlling purposes. In order to illustrate how to apply one of these techniques within the control software of a real robot, Section 3 analyzes the dynamic and sensors models of one of our robots, explains the general and efficient algorithm that has been adapted to use in the localization module of the robot control software, analyzes the performance of this algorithm under simulated and real data, and compares its performance against some other applicable OOS techniques. Finally, some conclusions are presented in Section 4.

## 2. State of the Art

In this section we analyze the different OOS filters for non-linear systems that haven been developed during the last years as well as the autonomous robotic and tracking examples that are found in the literature and that already consider the OOSP.

### 2.1. OOS Algorithms for Non-Linear Systems

The algorithms analyzed in this section are those that can estimate the location ***x***_*t_k_*_ of a system at time *t_k_* given (1) the measurements ***z***_*s,t_i_*_ provided by *s* = 1 : *S* different sensors at different time stamps *t_i_*; and (2) the probability models *p*(***x***_*t*_*k*__*|****x***_*t*_*i*__, ***u***_*t_k_,t_i_*_) and *p*(***z***_*s,t_k_*_|***x***_*t*_*k*__) that establish that the location ***x***_*t*_*k*__ at *t_k_* probabilistically depends on the location ***x***_*t*_*i*__ at a previous time step *t_i_* and on the control signal ***u***_*t*_*k*_*,t*_*i*__ applied between *t_i_* and *t_k_*, and that the measurement ***z***_*s,t*_*k*__ probabilistically depends on the location ***x***_*t*_*k*__. In our analysis, we also include those OOS algorithms whose dynamic behavior is only modeled as *p*(***x***_*t*_*k*__*|****x***_*t*_*i*__) (*i.e.*, without control signals), because its extension to control systems is not difficult, and those systems that consider the control signal are more general that those systems that don’t.

One important type of system that fulfills those requirements, and which can be used to model different types of autonomous mobile robots, is the group of *non-linear systems with Gaussian probabilistic density functions* that can be modeled with the expressions in Equation (1), where *N_a_*(***b***, ***C***) represents the normal distribution of variable ***a*** with mean ***b*** and covariance ***C***, *f*(*·*) and *h_s_*(*·*) are the transition and measurement functions that capture the dependency relationships among the variables, and ***Q***_*t*_*k*_*,t*_*i*__ and ***R***_*s,t*_*k*__ stand for the covariance matrices in the transition and measurement models.

(1) $$\begin{array}{c} p\left(x_{t_{k}}|x_{t_{i}},\;u_{t_{k},t_{i}}\right)=N_{x_{t_{k}}}\left(f\left(x_{t_{i}},u_{t_{k},t_{i}},t_{k},t_{i}\right),Q_{t_{k},t_{i}}\right)\\ p\left(z_{s,t_{k}}|x_{t_{k}}\right)=N_{z_{s,t_{k}}}\left(h_{s}\left(x_{t_{k}},t_{k}\right),R_{s,t_{k}}\right)\;\;\;\;\;\forall \;s=1:S \end{array}$$

An equivalent representation of the system is presented in Equation (2), where ***ν***_*t*_*k*_*,t*_*i*__ and ***μ***_*s,t*_*k*__ are random variables with zero mean and covariances ***Q***_*t*_*k*_*,t*_*i*__ and ***R***_*s,t*_*k*__ that represent the additive noise of the transition/measurements non-linear models *f*(*·*) and *h_s_*(*·*).

(2) $$\begin{array}{lll} x_{t_{k}} & = & f\left(x_{t_{i}},u_{t_{k},t_{i}},t_{k},t_{i}\right)+\nu_{t_{k},t_{i}}\\ z_{s,t_{k}} & = & h_{s}\left(x_{t_{k}},t_{k}\right)+\mu_{s,t_{k}}\;\;\;\;\;\forall \;s=1:S \end{array}$$

In order to estimate the value of ***x***_*t*_*k*__ for the non-linear problem presented in either Equation (1) or Equation (2), we can approximate *p*(***x***_*t*_*k*__*|****z***_1:*S,t*_0:*k*__, ***u***_*t*_0_:*t*_*k*__) as *N*_*x*_*t*_*k*___ (***x̂***_*t*_*k*_*|t*_*k*__, ***P***_*t*_*k*_*|t*_*k*__) using the Linearized Kalman or Information Filters (LKF/LIF), the Extended Kalman or Information Filter (EKF/EIF), the Unscented Kalman Filter (UKF), or the Ensemble Kalman Filter (EnKF) [2,3,22,23]. When the noise and or relationships are not necessarily Gaussian and/or linear (*i.e.*, when *p*(***x***_*t*_*k*__*|****x***_*t*_*i*__, ***u***_*t*_*k*_,*t*_*i*__) and *p*(***z***_*s,t*_*k*__*|****x***_*t*_*k*__) are generic probability density functions), the estimation problem can be solved using sequential Monte Carlo techniques, such as the Sampling Importance Resampling PF (SIR, [4]) or the Unscented PF (UPF, [24]) that approximate *p*(***x***_0:*t*_*k*__|***z***_1:*S,t*_0:*k*__, ***u***_*t*_0_:*t*_*k*__) using the point-mass distribution $\sum_{j=1}^{N}w^{\left(j\right)}\delta \left(x_{t_{0}:t_{k}}-x_{t_{0}:t_{k}}^{\left(j\right)}\right)$, or the Marginal PF (MPF, [25]) that approximates *p*(***x***_*t*_*k*__*|****z***_1:*S,t*_0:*k*__, ***u***_*t*_0_:*t*_*k*__) using the point-mass distribution $\sum_{j=1}^{N}w^{\left(j\right)}\delta \left(x_{t_{k}}-x_{t_{k}}^{\left(j\right)}\right)$.

The basic formulations of the LKF/LIF, EKF/EIF, UKF, EnKF, and PF cannot deal with the OOSP. Therefore, new OOS versions of these filters have been developed in the last decade. We briefly describe them in the following paragraphs, referring to those OOS algorithms that appear in the same paper as others by the paper number and its habitual nomenclature in the OOS literature (for instance, [5]-A1 represents the OOS algorithm A1 by Bar-Shalom [5]).

- *OOS Linearized KF/IF* ([26]): Linearizing the system models makes possible the direct use of the OOS KF/IF in the linearized system. Although this approach is applied with success in [26], where the OOS 1-step lag [5]-A1 KF is used for tracking autonomous vehicles with visual information only delayed 1 time step, it can produce erroneous results when the robot dynamic and sensorial models have strong non-linearities.
- *OOS Extended KF/IF* ([27–31]): They extend some OOS KF/IF to make them deal with the non-linearities of the problem:
  - [27,28]-EB1 present the extension of the retrodiction OOS KF in [32]-B1 to the non-linear case. This OOS EKF can work with non-linear measurement functions *h_s_*(***x***_*t*_*k*__*, t_k_*), although its transition function *f*(***x***_*t*_*i*__*,****u***_*t*_*k*_,*t*_*i*__*, t_k_, t_i_*) has to be linear (***F***_*t*_*k*_,*t*_*i*__***x***_*t*_*i*__ + ***u***_*t*_*k*_,*t*_*i*__), due to the retrodiction (backward propagation) operation used in [32]-Bl. Besides, [27,28]-EB1 only deals with the 1-step lag OOSP and can produce erroneous results when the robot dynamic and sensorial models have strong non-linearities.
  - [29] study the performance of extended versions of the retrodiction OOS KF in [33]-Al1 and [33]-Bl1, and the forward OOS KF on [34]&[29]-FPFD in a system with linear transition and non-linear measurement models. Although the linearity of the transition model is a requirement of the retrodiction step only in [33]-Al1 and [33]-Bl1, [34]&[29]-FPFD will only work properly if the non-linear transition model can be used to calculate properly the mean value of ***x***_*t*_*k*__ = *f*(***x***_*t*_*i*__, ***u***_*t*_*k*_,*t*_*i*__*, t_k_, t_i_*) for any Δ*t* = *t_k_* − *t_i_*. Besides, the Extended OOS KFs presented in [29], and called [29]-EAl1, [29]-EBl1, [29]-EFPFD hereafter, can produce erroneous results when the robot dynamic and sensorial models have strong non-linearities.
  - [30,31]-EIFAsyn extends the forward OOS IF presented in [30,31]-IFAsyn to work with systems with linear and/or non-linear transition and sensorial models. Besides, in order to overcome the difficulties that the former non-linear OOS algorithms face in systems with strong non-linearities, it distinguish two types of non-linear sensors: those whose information needs to be recalculated when older measurements arrive at the localization module and those whose information do not need to be recalculated.
- *OOS Unscented KF* ([35]): The OOS Unscented KF in [35] combines the retrodiction step of the OOS KF in [5] with the measurement update of the Unscented KF [22]. It requires a linear transition model (due to the retrodiction step) and it only deals with the 1-step lag OOSP.
- *OOS Ensemble KF* ([36]): The OOS Ensemble KF in [36] modifies the Ensemble KF [23] to let it work with N-step lag multisensor OOSP, by fusing the estimates obtained by *S* independent Ensemble KFs, each of them including a (1) backward propagation step that calculates the location values at the measurement time stamp linearly interpolating the location values of the previous and posterior time steps and (2) a new set of assimilation operations that let it deal with the delays of the measurements.
- *OOS PF* ([37–45]): They modify the non-OOS SIR, UPF and MPF to let them work with the OOS measurements ***z***_*s,t*_*m*__ only in systems with Gaussian probability models (Equation (1) or Equation (2)). That is, although the basic versions of these PFs do not require Gaussian probability models, its OOS counterparts, described next, need it due to different assumptions made during their development:
  - The OOS SIR PFs ([37–40] and [42]-A) and the OOS UPF ([41]) update the weights of the particles making $w^{\left(j\right)}\propto w^{\left(j\right)}p\left(z_{s,t_{m}}|x_{t_{m}}^{\left(j\right)}\right)$. The value of $x_{t_{m}}^{\left(j\right)}$ is either (1) the value of the location of the j-th particle at time *t_m_* when the PF has already associated a measurement for the time stamp of the new ***z***_*s,t*_*m*__; or (2) the value sampled from a probability distribution $q(x_{t_{m}}|x_{t_{a}}^{\left(j\right)},x_{t_{b}}^{\left(j\right)},z_{s,t_{m}})$, different for each type of PF, that takes into account the values of the particles at *t_a_* and *t_b_*, where *t_a_* and *t_b_* are the closest lower and higher time stamps to *t_m_* of already assimilated measurements. The sampling distribution in [37–39], $q(x_{t_{m}}|x_{t_{a}}^{\left(j\right)},x_{t_{b}}^{\left(j\right)})$, does not consider the current measurement and exploits the properties of a Gaussian linear transition model. The value of $x_{t_{m}}^{\left(j\right)}$ in [40] is obtained linearly interpolating the location values of $x_{t_{a}}^{\left(j\right)}$ and $x_{t_{b}}^{\left(j\right)}$. The sampling distribution in [41], $q_{\mathrm{UKF}}\left(x_{t_{m}}|x_{t_{a}}^{\left(j\right)},x_{t_{b}}^{\left(j\right)},z_{s,t_{m}}\right)$, is obtained with an UKF whose prediction step has the same linearity requirement as the $q\left(x_{t_{m}}|x_{t_{a}}^{\left(j\right)},x_{t_{b}}^{\left(j\right)}\right)$ in [37–39] and whose update step incorporates ***z***_*s,t*_*m*__ using the unscented transformation. Finally, the sampling distribution in [42]-A, $q_{\mathrm{EKF}}\left(x_{t_{m}}|x_{t_{a}}^{\left(j\right)},x_{t_{b}}^{\left(j\right)}\right)$, is obtained with an EKF with a prediction step from *t_a_* to *t_m_* that uses the system Gaussian transition model, and an update step that treats the transition from *t_m_* to *t_b_* as a measurement.
  - The OOS MPFs ([42]-B# and [43–45]) update the weights of the particle for the delayed measurements making $w^{\left(j\right)}\propto p\left(z_{s,t_{m}}|x_{t_{m}}^{\left(j\right)},z_{1:s,t_{0}:t_{k}}\right)w^{\left(j\right)}$. Besides, they update the values of the non-delayed measurements with the usual SIR step, which creates PFs that acs as SIR when *t_m_* ≥ *t_k_* and as MPFs when *t_m_* < *t_k_*. However, as the SIR step is equivalent to the MPF step with transition prior [25], we can classify [42]-B# and [43–45] as purely MPFs. The MPFs in [42] obtain $p\left(z_{s,t_{m}}|x_{t_{m}}^{\left(j\right)},z_{1:s,t_{0}:t_{k}}\right)$ exploiting the property that this probability is dependent on the smoothed density $p\left(x_{t_{m}}|x_{t_{m}}^{\left(j\right)},z_{1:s,t_{0}:t_{k}}\right)$, which can be approximated using a Fixed-Point Extended Kalman Smoother ([42]-B1), a Fixed-Point Unscented Smoother ([42]-B2) or a Fixed-Point Particle Smoother ([42]-B3). The $p\left(z_{s,t_{m}}|x_{t_{m}}^{\left(j\right)},z_{1:s,t_{0}:t_{k}}\right)$ in [43] is approximated by $p\left(z_{s,t_{m}}|x_{t_{m}}^{\left(j\right)}\right)$, obtaining $x_{t_{m}}^{\left(j\right)}$ with the retrodiction step in [5]. Finally, the MPFs in [44] and [45] improve the efficiency of the MPFs in [42] using some additional mechanisms to select which of the delayed measurements should be assimilated.

The main characteristics of the OOS non-linear filters are summarized in Table 1. The first column (*ALG*) identifies the algorithm, the second (*Group*) the group they belong to, the third (*Extending*) the algorithm they are extending/modifying, and the fourth (*Support*) the core idea or distribution function that lets the algorithm obtain the location associated to the time stamp of the delayed measurement and assimilate the measurement information into the current location estimate. The fifth (*Trans. Model*) and sixth (*Meas. Model*) show which types of Gaussian Transition and Measurement Models the filters are restricted to: L stands for linear, NL for Non-Linear, and Lzd for linearized. The seventh column (*OOSP*) identifies if they are prepared to deal with the 1-step lag or N-step lag OOSP. Finally, the eighth column (*Extras*) shows other important additional information: [30,31]-EIFAsyn optionally recalculate the sensorial information of some of the already assimilated measurements whose time stamp is bigger than the just arrived one to avoid the problems associated to systems with strong non-linearities, the MCMC step in [38,39] increments the diversity of the old history values to reduce the problems associated with particles depletion, and the check diversity mechanisms in [42]-B# and [44,45] are used to decide whether an OOS ***z***_*s,t*_*m*__ should be assimilated or not.

An analysis of the table shows that the OOS non-linear algorithms that can be used for systems with linear and/or nonlinear systems with measurements delayed more than 1-step lag are: the OOS EIF [30,31]-EIFAsyn, the OOS EnKF [36], the OOS SIR [40], and the OOS MPFs [42]-B#, [44] and [45]. The use of a linear interpolation operation to obtain the location at the time stamp of a delayed measurement in [36] and [40] reduces the applicability of these algorithms to those systems with slow dynamics or whose position between two time steps can be approximated by the line that joins the positions of the previous and posterior time-steps. The remaining techniques are equally general, although the computational overload associated to the OOS EnKF (penalized further in the multisensorial case by the use of as many EnKFs as sensors) and OOS MPFs (penalized further by the operations of the fix-point smoothers), makes the OOS EIF [30,31]-EIFAsyn the most efficient solution, when there are some linear or weakly non-linear measurement models that make the filter avoid the recalculation of the sensorial information associated to them.

### 2.2. Autonomous Robot Control and Tracking Systems that Deal with the OOSP

To the best of the authors’ knowledge, there is not any published localization module that forms part of a control system of an autonomous mobile robots that deals with the OOSP using an OOS algorithm, instead of throwing the delayed measurements (easiest and more frequent approach), delaying the operations [11], or re-starting the filter from the time stamp of each arriving measurement [12,13].

However, the majority of the OOS non-linear filters analyzed in the previous section are used to track the location of mobile objects. Based on the information contained in the robot location ***x****_t_* estimated by the OOS non-linear filter, the tracking problems (and the OOS filters) can be organized as:

1. *Pose* (*p^x^, p^y^*) + *Velocity* (*v^x^, v^y^*) *Estimation Problems* with a linear dynamic velocity constant model. The OOS LKF [26], OOS EKF in [27–29], OOS UKF in [35], OOS EnKF in [36], OOS PFs in [37–39,43] have been tested against this type of model. Besides, the OOS EKF [29]-EA1 and [29]-EFPFD are implemented successfully inside an automative pre-crash system [46].
2. *Pose* (*p^x^, p^y^*) *+ Velocity* (*v^x^, v^y^*) *+ Orientation* (*p^θ^*) *Estimation Problems*, where the dynamic model is the non-linear coordinated turning model in [47]. The PFs in [42,44,45] have been tested against this type of model. The OOS UPF [41], whose $q_{\mathrm{UKF}}\left(x_{t_{m}}|x_{t_{a}}^{\left(j\right)},x_{t_{b}}^{\left(j\right)},z_{t_{m}}\right)$ requires a linear transition model, is tested against this type of problem too, because the non-linear transition model *f*(***x***_*t*_*i*__, ***u***_*t*_*k*_,*t*_*i*__) of the coordinated turning model can be factored as ***F***_*t*_*k*_,*t*_*i*__ (***x***_*t*_*k*__)***x***_*t*_*k*__. Besides, two reduced version of this estimation problem, consisting on estimating only the pose (*p^x^, p^y^*) and orientation (*p^θ^*) with a non-linear dynamic model are used to analyze the performance of the OOS EIF [30,31]-EIFAsyn and the OOS SIR in [40].

From the point of view of a tracking system both problems are equally interesting, although the second is more difficult due to (1) the non-linearities in the transition model and to (2) the discontinuity between 0 and 2*π* (or between −*π* and *π*) associated with the orientation *p^θ^*. From the point of view of a control system whose actions depend on the location estimates, the second type of problem is more complete, because estimating also the orientation usually allows a finer control of the robot.

### 2.3. Suggested OOS Algorithms for the Location Module of an Autonomous Robot Control System

Based on the previous analysis, when the robot dynamics are modeled with non-linear expressions in the localization module of the control system of an autonomous mobile robot, we suggest to select those algorithms that have an inherent complete non-linear support ([36,40,42,44,45] and [30,31]-EIFAsyn) and/or whose performance has been tested already with non-linear modeled systems ([40–42,44,45] and [30,31]-EIFAsyn). Besides, we consider that the OOS EIF [30,31]-EIFAsyn is especially interesting in those cases where a low computational load is required in the localization module, because it avoids the computational burden associated to the OOS PFs [40–42,44,45] and OOS EnKF [36].

When the robot dynamics can be modeled with linear expressions, any of the techniques with linear and/or non-linear dynamic model support can be applied. However, special care should be taken with the OOS EKFs that by default do not consider that those sensors with strong non-linearities can require to have the information recalculated when measurements with older time stamps arrive later than the ones associated to them that have been already assimilated.

## 3. A Case Study

This section illustrates the benefits of using one of these OOS techniques by implementing an adapted version of [30,31]-EIFAsyn in the localization module of the control software of one of our robots. Additionally, its non-linear dynamics and combination of linear, weakly non-linear and strongly non-linear sensors let us show the importance of analyzing the properties of all the sensors to minimize the computational requirements while maintaining the performance of the selected filter. Finally, this section also includes a performance comparison of the selected technique against a subset of the previously recommended filters.

### 3.1. The Robot Dynamic and Sensorial Models

In this section we present the dynamic and sensorial models associated to the localization module of the autonomous mobile robot that we will use in our experiments. Besides, the constants and covariances of the models are presented in Section 3.3.1, and its Jacobians in the Appendix.

**Figure 2.**
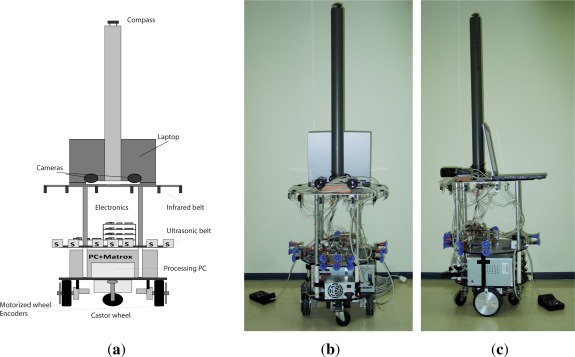
Robot. (**a**) Schema; (**b**) Frontal View; (**c**) Lateral View.

The robot, represented in Figure 2, is equipped with two motorized wheels (independently controlled by two DC drives and placed, separated at *b* distance, under the lower robot platform) and two castor wheels (placed in the front and back of the same platform). The dynamic behavior of a robot with this arrangement, which lets the robot rotate around its Z-axis with an angular speed dependent on the control speed applied to each wheel, is going to be modeled as a non-linear system. The sensorial devices of the robot used to estimate the robot location are: two encoders attached to the motorized wheels that provide information about the displacement of the wheels, a magnetic compass that provides information of the robot orientation, and an ultrasonic belt that provides information of the robot distance to known landmarks. These three types of sensors are going to be modeled with linear and non-linear models. Finally, it is worth noting that the robot is also equipped with an stereoscopic visual system and an infrared belt, whose information is only used in the module in charge of updating the map information.

#### 3.1.1. Robot Dynamics

The location *x_t_* of the robot at each time step consists of five components: the robot pose $\left(p_{t}^{x},p_{t}^{y}\right)$, its orientation $\left(p_{t}^{\theta }\right)$, and the linear and angular displacements of the robot $\left(\Delta_{t}^{l},\Delta_{t}^{\theta }\right)$. The control signal ***u****_t_* has two components $\left(u_{t}^{L},u_{t}^{R}\right)$ that represent the velocity applied to the Left and Right motorized wheels.

The dynamics of our robot can be modeled with different types of expressions [48,49]. The selected model, whose transition function *f*(*·*) is presented in Equation (3), decomposes the robot movement in a rotation $\left(\Delta_{t}^{\theta }\right)$ followed by a linear displacement $\left(\Delta_{t}^{l}\right)$, without considering the thickness of the motorized wheels or the rotations that occur while the robot is displacing. In the model, the linear displacement $\left(\Delta_{t+1}^{l}\right)$ is obtained as the mean displacement obtained by both motorized wheels moving at speeds $\left(u_{t}^{L},u_{t}^{R}\right)$ during *dt* seconds, while the angular displacement $\left(\Delta_{t+1}^{\theta }\right)$ depends on the difference of the displacement of both wheels and the distance (*b*) that exists between them. Finally, it is important to highlight the fact that the predicted orientation $\left(p_{t+1}^{\theta }\right)$ is obtained incrementally, and therefore its values can be out of one of the usual angular range (such as [0, 2*π*) or [−*π, π*)). Therefore, in order to keep the values in a given range, it is advisable, although not necessary, to put them into range after the prediction.

(3) $$x_{t+1}=\left(\begin{array}{c} p_{t+1}^{x}\\ p_{t+1}^{y}\\ p_{t+1}^{\theta }\\ \Delta_{t+1}^{l}\\ \Delta_{t+1}^{\theta } \end{array}\right)=f(x_{t},\;u_{t},\;\mathrm{dt})=\left(\begin{array}{l} p_{t}^{x}+\Delta_{t}^{l}\;\text{cos}\;(p_{t}^{\theta }+\Delta_{t}^{\theta })\\ p_{t}^{y}+\Delta_{t}^{l}\;\text{sin}\;(p_{t}^{\theta }+\Delta_{t}^{\theta })\\ p_{t}^{\theta }+\Delta_{t}^{\theta }\\ \mathrm{dt}*[u_{t}^{R}+u_{t}^{L}]/2\\ \mathrm{dt}*[u_{t}^{R}-u_{t}^{L}]/b \end{array}\right)$$

The covariance matrix *Q*_*t*+1*,t*_ of the white noise added to the transition function is presented in Equation (4), and depends of the square of the elapsed time *dt*. Note that this *Q*_*t*+1*,t*_ makes the noise accumulate directly in the displacement variables and indirectly in the pose and orientation.

(4) $$Q_{t+1,t}={\mathrm{dt}}^{2}*\left(\begin{array}{ccccc} 0 & 0 & 0 & 0 & 0\\ 0 & 0 & 0 & 0 & 0\\ 0 & 0 & 0 & 0 & 0\\ 0 & 0 & 0 & {\mathrm{var}}_{\Delta^{l}} & 0\\ 0 & 0 & 0 & 0 & {\mathrm{var}}_{\Delta^{\theta }} \end{array}\right)$$

#### 3.1.2. Compass

**Figure 3.**
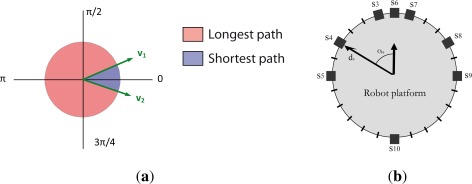
Sensor Models. (**a**) Orientation; (**b**) Ultrasonic belt.

The robot is equipped with an electronic compass that provides information (*z*_1*,t*+1_) of the robot orientation $\left(p_{t+1}^{\theta }\right)$. However, due to the discontinuity and periodicity of the angular data, the measurement function *h*_1_(*·*) should receive a special treatment, which takes into account if the angular discontinuity forms part of the shortest or longest path that exist, according to Figure 3(a), between the compass measurement ***z***_1,*t*+1_ and the robot orientation $\left(p_{t+1}^{\theta }\right)$. When that happens, and in order to make the robot location comparable to the compass measurement, the $p_{t+1}^{\theta }$ value should be incremented/decremented for 2*π* radians. The measurement function *h*_1_(*·*) that represents this behavior is presented in Equation (5). The first expression is for the case where the discontinuity is in the shortest path and ***z***_1,*t*+1_ is *v*_1_ and $p_{t+1}^{\theta }$ is *v*_2_. The second expression is for the case where the discontinuity is in the shortest path and ***z***_1,*t*+1_ is *v*_2_ and $p_{t+1}^{\theta }$ is *v*_1_. And the last expression is for the case where the discontinuity is not in the shortest path.

(5) $$z_{1,t+1}=\{\begin{array}{ll} p_{t+1}^{\theta }-2\pi & \text{if}\;z_{1,t+1}\in [0,\pi )\;\wedge |z_{1,t+1}-p_{t+1}^{\theta }|\;>\pi \\ p_{t+1}^{\theta }+2\pi & \text{if}\;z_{1,t+1}\in [\pi ,2\pi )\;\wedge |z_{1,t+1}-p_{t+1}^{\theta }|\;>\pi \\ p_{t+1}^{\theta } & \text{otherwise} \end{array}$$

The covariance matrix ***R***_1_*_,t_*_+1_ of the white noise added to the compass measurement function, presented in Equation (6), is the same for every compass measurement.

(6) $$R_{1,t+1}=\left({\mathrm{var}}_{\mathrm{compass}}\right)$$

#### 3.1.3. Encoders

The robot is also equipped with two incremental encoders that can be used to obtain information about the displacement of each motorized wheel. This displacement, represented as $z_{2,t+1}^{N_{L}}$ and $z_{2,t+1}^{N_{R}}$ for the Left and Right wheel, is linearly related, as the encoder measurement model in Equation (7) captures, to the robot linear and angular displacements ( $\Delta_{t+1}^{l}$ and $\Delta_{t+1}^{\theta }$).

(7) $$z_{2,t+1}=\left(\begin{array}{c} z_{2,t+1}^{N_{L}}\\ z_{2,t+1}^{N_{R}} \end{array}\right)=\left(\begin{array}{c} \Delta_{t+1}^{l}-\Delta_{t+1}^{\theta }*b/2\\ \Delta_{t+1}^{l}+\Delta_{t+1}^{\theta }*b/2 \end{array}\right)$$

The covariance matrix ***R***_2_*_,t_*_+1_ of the white noise added to the encoder measurement function, presented in Equation (8), depends on the square of *dt* and considers that the errors of the encoders are independent.

(8) $$R_{2,t+1}={\mathrm{dt}}^{2}*\left(\begin{array}{cc} {\mathrm{var}}_{N_{L}} & 0\\ 0 & {\mathrm{var}}_{N_{L}} \end{array}\right)$$

#### 3.1.4. Ultrasonic System

Finally, the robot is also equipped with a belt of 8 ultrasonic sensors numbered from s = 3 to s = 10 (as s = 1 and s = 2 are already associated to the compass and the encoders), distributed around the upper robot platform as Figure 3(b) shows, able to measure the distance of objects placed between 0.2 and 2.0 m. This distance measurements can be used to determine the robot pose $\left(p_{t+1}^{x},p_{t+1}^{y}\right)$ when the robot is placed on an environment with known walls, corners and columns. In order to establish the relation of the robot and landmarks poses, we use the relations proposed in [50], where:

- The distance between a *wall* and the sensor can be calculated as the distance between the normalized line (*A_obj_x* + *B_obj_y* + *C_obj_* = 0, with $A_{\mathrm{obj}}^{2}+B_{\mathrm{obj}}^{2}=1$) that defines where the wall is placed and the sensor pose (*x, y*), as far as the orientation of the sensor and the wall fulfill the constraints imposed by the sonar directivity.
- The distance between a *corner* and the sensor can be calculated as the distance between the corner pose $\left(p_{\mathrm{obj}}^{x},\;p_{\mathrm{obj}}^{y}\right)$ and the sensor pose (*x, y*), as far as the orientation of the sensor and of the line that joins the sensor with the corner fulfill the constraints imposed by the sonar directivity.
- The distance between a *column* and the sensor can be calculated as the distance between the circle of radius (*r_obj_*) and center $\left(p_{\mathrm{obj}}^{x},\;p_{\mathrm{obj}}^{y}\right)$ that represents the column, and the sensor pose (*x, y*), as far as the orientation of the sensor and of the line that joins the sensor with the center of the column fulfill the constraints imposed by the sonar directivity.

In order to relate the robot pose $\left(p_{t+1}^{x},\;p_{t+1}^{y}\right)$ directly with the distance provided by the sensors, we can use the relation between the robot pose and sensor disposition (represented by the distance *d_s_* and orientation *α_s_* according to Figure 3(b)):

(9) $$\begin{array}{l} x=p_{t+1}^{x}+d_{s}\;\text{cos}\left(p_{t+1}^{\theta }+\alpha_{s}\right)\\ y=p_{t+1}^{y}+d_{s}\;\text{sin}\left(p_{t+1}^{\theta }+\alpha_{s}\right) \end{array}$$

Taking into account all these factors, the non-linear function that models the behavior of each ultrasonic sensor (*s* ∈ [3 : 10]) with respect to a map of given landmarks are represented in Equation (10), where *obj* represent the detected object and its type. In order determine which object has been detected, we run the model, taking into account the sonar orientation and directivity for all the objects of the map and select among all the possible identified objects that are not occluded by others the one that will produce a shorter distance signal, because it is the one that has a higher chance of having reflected the sonar signal.

(10) $$z_{s,t+1}=\{\begin{array}{ll} \left|A_{\mathrm{obj}}\left[p_{t+1}^{x}+d_{s}\;\text{cos}(p_{t+1}^{\theta }+\alpha_{s})\right]+B_{\mathrm{obj}}\left[p_{t+1}^{y}+d_{s}\;\text{sin}(p_{t+1}^{\theta }+\alpha_{s})\right]+C_{\mathrm{obj}}\right| & |\;\mathrm{obj}=\mathrm{wall}\\ \sqrt{{\left[p_{t+1}^{x}+d_{s}\;\text{cos}(p_{t+1}^{\theta }+\alpha_{s})-p_{\mathrm{obj}}^{x}\right]}^{2}+{\left[p_{t+1}^{y}+d_{s}\;\text{sin}(p_{t+1}^{\theta }+\alpha_{s})-p_{\mathrm{obj}}^{y}\right]}^{2}} & |\;\mathrm{obj}=\mathrm{corner}\\ \sqrt{{\left[p_{t+1}^{x}+d_{s}\;\text{cos}(p_{t+1}^{\theta }+\alpha_{s})-p_{\mathrm{obj}}^{x}\right]}^{2}+{\left[p_{t+1}^{y}+d_{s}\;\text{sin}(p_{t+1}^{\theta }+\alpha_{s})-p_{\mathrm{obj}}^{y}\right]}^{2}}-r_{\mathrm{obj}} & |\;\mathrm{obj}=\mathrm{column} \end{array}$$

The covariance matrix ***R***_*s,t+1*_ with *s* ∈ [3 : 10] of the white noise added to the ultrasonic distance measurement function, presented in Equation (11), is the same for every sonar and distance measurement.

(11) $$R_{s,t+1}=\left(\mathrm{va}r_{\mathrm{sonar}}\right)$$

#### 3.1.5. Models Summary

In short, the expressions used to model the robot behavior have the following characteristics:

- The dynamic/transition model is non-linear. Besides, the angular location $\left(p_{t+1}^{\theta }\right)$ has a limited and periodic representation. The influence of this fact in the correct behavior of the filters will be analyzed in the following sections.
- The compass model is also non-linear due to the *±*2*π* correction term that appears, dependent on the value of ***z***_1*,t*+1_ and $p_{t+1}^{\theta }$, in certain cases as a consequence of the discontinuity and periodicity of the angular data.
- The two encoders grouped together in ***z***_2*,t*+1_ are modeled using a unique linear expression and covariance matrix.
- The behavior of the sensor that combines the measurement of each ultrasonic sensor *s ∈* [3 : 10] and a map of objects is represented with a non-linear model.

That is, we are going to estimate the location of a robot using a non-linear transition model, and linear and non-linear measurement models.

### 3.2. EIFAsyn: The Selected Estimator for the Localization Module

In this section, we present an adapted version of EIFAsyn, the OOS EIF that consists of sets of EKF predictions, EIF updates and projections between the state and information space [30,31], that has been slightly modified in order to (1) consider the peculiarities of the angular data and to (2) include a validation step to decide before assimilating the sensorial information, whether the measurement is corrupted or not.

**Figure 4.**
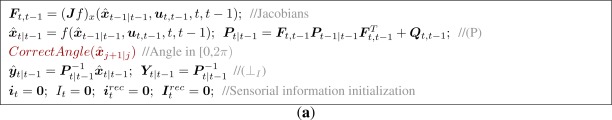

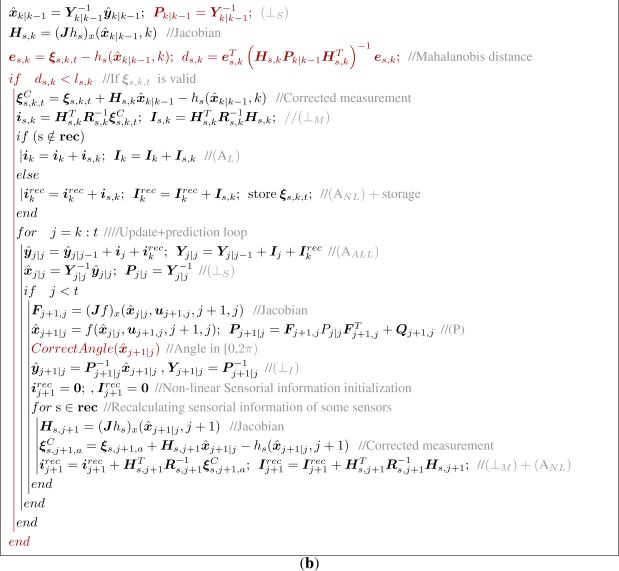
Prediction and Measurement Update Steps of the adapted EIFAsyn. (**a**) Prediction step from *t* − 1 to *t*; (b) Update step for ***ξ****_s,k,t_* (measurement of sensor *s* with time stamp *k* arriving at *t*).

The two main steps of this version of EIFAsyn, prediction (carry out to make the filter estimate the state of the next time step) and measurement update (performed when any measurement is arrived), are presented in Figure 4, where ***ξ****_s,k,t_* represents, maintaining the nomenclature used in [30,31], the measurement ***z****_s,k_* taken by sensor *s* at time *k* that arrives at the localization module at *t*. Besides, ***x̂****_k|k_* and ***P****_k|k_* stand for the mean and covariance of the location estimated with all the measurements that have been assimilated so far, ***ŷ****_k|k_* and ***Y****_k|k_* for the information of the estimated location and its covariance, ***i****_k_* and ***I****_k|k_* for the accumulated sensorial information and accumulated sensorial information covariance of the sensors whose information does not need to be recalculated, and $i_{k}^{\mathrm{rec}}$ and $I_{k|k}^{\mathrm{rec}}$ for the accumulated sensorial information and accumulated sensorial information covariance of the sensors whose information has to be recalculated. Finally, the additional operations that do not appear in the original version of EIFAsyn are presented in red to make them easily identifiable.

The EIFAsyn prediction step (Figure 4(a)) is the usual prediction step of the EKF (Jacobians + (P)), followed by the additional operation that corrects the angular data, a projection of the location and its covariance into to information space (*⊥_I_*), and an initialization of the sensorial information variables.

The EIFAsyn update step (Figure 4(b)) consists of several steps. It starts with a validation step that calculates the Mahalanobis distance *d_s,k_* (which weights the discrepancy between the measurement and predicted measurement with the inverse of the predicted measurement covariance) and checks if *d_s,k_* is under a threshold *l_s_* selected to ensure that the test only rejects valid measurements with a selected probability [15,51]. To assimilate the valid measurements, the update step projects the corrected measurement $\xi_{s,k,t}^{C}$ into the information space (*⊥_M_*), accumulates their values to the remaining sensorial information associated to the same time step (*A_L_* or *A_NL_*), and optionally stores the measurement. Afterwards, it starts an assimilation-prediction loop, where all the sensorial information at each time step is accumulated into the predicted information values (*A_ALL_*), projected into the state space (*⊥_S_*), predicted (P), corrected, and projected again into the information space (*⊥_I_*). Additionally, the algorithm recalculates the sensorial information of those sensors that require this operation (*⊥_M_* + *A_NL_*).

The recalculation of the information of sensor *s* is required when ***H****_s,j_*_+1_ ***x̂**_j_*_+1_*_|j_* − *h_s_*(***x̂**_j_*_+1_*_|j_*, *j* + 1), the term that corrects the measurements ***ξ****_s,j_*_+1_*_,a_*, changes its value significantly with the changes that occur to ***x̂****_j_*_+1_*_|j_* during the assimilation-prediction loop. For the linear sensors, the recalculation is never necessary because ***H****_s,j_*_+1_***x̂****_j_*_+1_*_|j_* − *h_s_*(***x̂****_j_*_+1_*_|j_*, *j* + 1) = 0. Moreover, in that case $\xi_{s,k,t}^{C}=\xi_{s,k,t}$. For the non-linear sensors, the recalculation is unnecessary when the changes are negligible and it is required when the changes are abrupt.

Therefore it is necessary to study the behavior of the non-linear sensors before deciding to exclude them from the recalculation set. In our problem, we have to analyze the behavior of the:

- *Compass*: Given a compass measurement ***z***_1*,j*+1_, the value returned by the compass model (Equation (5)) changes abruptly depending on the position of the indetermination (0, 2*π*) with respect to the shortest path between ***z***_1*,j*+1_ and the estimated angle $p_{j+1}^{\theta }$, while the value of ***H***_1*,j*+1_ ***x̂***_*j*+1*|j*_ is always $p_{j+1}^{\theta }$ as ***H***_1_*_,j_*_+1_ (Equation (13)) remains unchanged. Therefore, when a new delayed measurement modifies the estimated value of $p_{j+1}^{\theta }$, placing it at the opposite side of the indetermination it was before the assimilation of the new measurement, there can be an abrupt change of *±*2*π* between the previous-assimilation and post-assimilation values of ***H***_1_*_,j_*_+1_***x̂****_j_*_+1_*_|j_* − *h*_1_(***x̂****_j_*_+1_*_|j_, j* + 1). As when the robot orientation is close to the indetermination, delayed measurements can easily change the values of $p_{j+1}^{\theta }$ to the opposite site, the already assimilated compass information needs to be recalculated when any delayed measurement arrives to avoid the erroneous operation of EIFAsyn.
- *Ultrasonic system*: Given a sonar measurement ***z****_s,j_*_+1_ with *s ∈* [3, 10], the value returned by the ultrasonic model (Equation (10)) and the value of ***H****_s,j_*_+1_***x̂****_j_*_+1_*_|j_* (Equation (13)) are smoothly changed with small modifications of the estimated robot pose $\left(p_{j+1}^{x},p_{j+1}^{y}\right)$. Therefore, when the robot pose estimate is slightly modified by the arrival of a given measurement, the previous-assimilation and post-assimilation values of ***H****_s,j_*_+1_***x̂****_j_*_+1_*_|j_*−*h_s_*(***x̂****_j_*_+1_*_|j_, j*+1) do not change significantly. Therefore, we can consider the exclusion of this sensor from the recalculation set.

The validity of both choices has been tested in multiple simulations. Before presenting their results in the following section, it is worth noting that the necessity of recalculating the sensorial information associated to some non-linear sensors is a requirement of EIFAsyn and of the OOS EKF (which can not be used for our problem due to the additional requirement of the linearity in the transition model), due to the direct relationship that exists between the EKF and EIF [3]. Besides, in the cases where the robot control software can support the computing overload of the OOS PF (that will not need to recalculate information associated to the already assimilated measurements) or OOS EnKF, those algorithms should also take into account the existence of the indetermination in the angular data when calculating the mean and covariance values of the robot locations based on the values of the particles or ensembles.

Finally, note that the additional step associated to the correction of the angle does not change the behavior of the algorithm significantly. Moreover, it is only a convenient modification. However, the step associated to the validation step, which is useful to reject those measurements that are not valid due to the malfunction of the sensors or corrupted during the communication through the network, has a bigger impact on the results of the algorithm. Moreover, as the validity of the measurements is only tested once using the location estimate in the test, the validity of the measurements that are in the limit can depend on the order of arrival of the measurements. However, as the analysis of the validation for the asynchronous version of IFAsyn shows [15], the influence of the order of arrival can be minimal when the filter is well-tuned.

### 3.3. Results Obtained with EIFAsyn

In this section we present the results obtained by EIFAsyn for the localization of our autonomous mobile robot with simulated and real data. The simulated experiments are set up to see which of the non-linear sensors require the recalculation of its measurements information when there are delayed data and what happens if the OOS data is not used. The real experiment shows if the localization module based on the OOS-EIF works properly when it is part of the control software of the actual robot.

**Figure 5.**
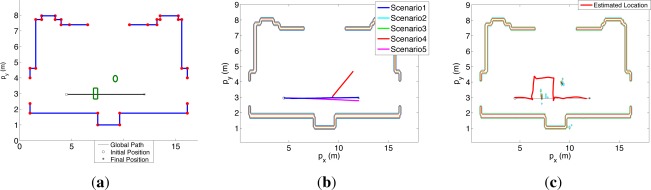
Experiments Setup. (a) Map Objects; (b) Simulated Experiment; (c) Real Experiment.

Figure 5(a) shows the setup of both experiments. The red dots represent all the known corners while the blue lines represent all the known walls. In the simulated experiment, the robot is placed in the initial position (o), oriented towards the final position (*), and controlled by applying equal speeds to its motorized wheels in order to move it around the angular indetermination [0, 2*π*]. Therefore, it follows the blue trajectory presented in Figure 5(b). In the real experiment, the robot is placed in the initial position (o) and required to go to the final position (*), unknowing that there are the square and round objects (marked in green in Figure 5(a)) in the hall. The control software of the robot where the OOS algorithm is embedded [52] makes the robot initially go towards the final position until it locates the unknown objects, updates the occupancy grid [53], and replans new trajectories to avoid the unknown objects locations. Therefore, the robot follows the red trajectory presented in Figure 5(c).

#### 3.3.1. Simulations

In order to analyze what happens if the delayed measurements are not used and which non-linear sensors require the recalculation of its information when there are delayed measurements, we run multiple simulations to generate all the sensors measurements every 0.1 s and run EIFAsyn, for the same generated data, considering 5 different scenarios: (1) using all the measurements without delay; (2) using all the measurements with delays in the compass data without recalculating the sonar information; (3) using all the measurements with delays in the sonar data recalculating the compass information; (4) using all the measurements with delays in the sonar data without recalculating the compass information; and (5) not using the delayed measurements of the compass and sonars. We use constant delays of 1 s in all the cases, *i.e.*, of 10 time-steps, in order to increment the challenge of the validation step (which only considers the already arrived measurements) of the adapted version of EIFAsyn presented in Section 3.2. The lack of delays in the first case makes EIFAsyn work as the EKF and therefore, the behavior of the filter in this scenario is considered the reference to be achieved by EIFAsyn in the remaining scenarios. The delays of the compass measurements in the second are used to see that the sonar information, arrived before the compass one in this case, does not have to be recalculated. Similarly, the delays of the sonar measurements in the third and fourth are used to see if the compass information, arrived before the sonar one in these scenarios, has to be recalculated or not. In other words, scenarios 2, 3, and 4 let us check if the non-linearities of the sonar and compass are soft or hard. Finally, the fifth case justifies the necessity of the OOS algorithm when all the compass and sonar information arrives delayed, as it happens in the real robot experiments.

As the non-linearity of the compass is due to the angular indetermination [0, 2*π*], we set up the simulated experiments to make it move around it, placing the robot initially oriented towards 0 rad, and applying the same control signals (10 cm/s) to both motorized wheels during the whole simulation. The simulated data is obtained with the same models that are incorporated in EIFAsyn, although as Table 2 shows the variances used to obtain the simulated data (fourth row) are lower than those used in EIFAsyn (and in the other filters under test in Section 3.4, fifth row). The difference in the variances is due to the following factors. On one hand, we originally set up the variance values in the simulations accordingly to the values of the transition and measurement noise levels that we have observed in our robot (third row). However, we have to significantly reduce the simulation value of *var*_Δ*^θ^*_, because the bigger real value significantly affects the simulated robot orientation and we want it to move with two equal fixed control signals (*i.e.*, without a feedback control loop) around the angular indetermination without diverging significantly from it. On the other, the variance values in EIFAsyn are bigger than the observed in order to reduce the effects of the non-linearities in the suboptimal EKF without delays.

**Figure 6.**
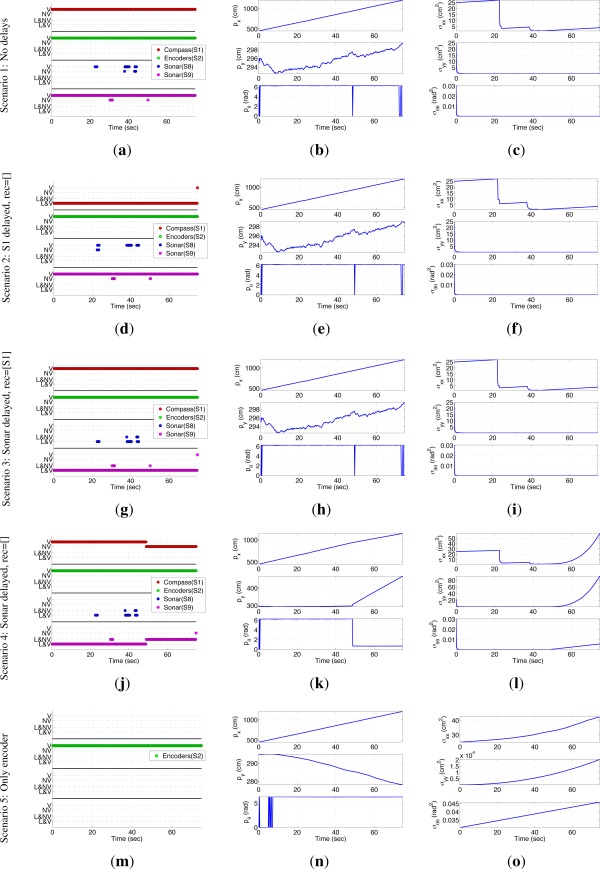
Simulated Experiments.

The results of EIFAsyn for the different setups and the values obtained during one of the simulations are summarized in the graphics presented in Figure 6.

The first column shows the behavior of the sensors and validation tests representing all the measurements received by EIFAsyn grouped by sensor (color) and status: *V*- non-delayed and valid, *NV*- non-delayed and non-valid, *L&NV*- delayed and non-valid, and *L&V*- delayed and valid. The second column shows the estimated value of the location (*p_x_, p_y_*) and orientation (*p_θ_*), and the third represents their covariances (*σ_xx_, σ_yy_, σ_θθ_*). In each row, we group the results of the same scenario. The estimated location (*p_x_, p_y_*) in the five cases is also presented in Figure 5(b) (the trajectories of the second and third case are occluded by the trajectory of the first, while the trajectories of the fourth and fifth scenario diverge). The graphics in the first column show that as a consequence of the robot orientation in the experiment setup S8 and S9 are the only sonars providing measurements. Moreover, S8 only gets a few measurements associated to the corners in the bottom U-shape of the hall, which provide both information associated to *p_x_* and *p_y_* and are responsible of the decrements in covariance of the *p_x_* state. The continuous stream of measurements of S9 are associated to the bottom walls of the hall, provide information about *p_y_* and are responsible of the the small covariance of the *p_y_* state. Finally, the continuous stream of measurements of the compass provides information about *p_θ_* and is responsible of the the small covariance of the *p_θ_* state.

At a first glance, the state and covariance graphics of the first, second and third row look really similar. That is, EIFAsyn obtains similar results when the data is non-delayed, when the compass data is delayed and the filter does not recalculate the non-delayed sonar information, and when the sonar data is delayed and the filter recalculates the non-delayed compass information. The sensor behavioral graphics look different because they encode both the existence of the delays and the results of the validation. If we compare only their validation status, we can observe that the same data is validated in the experiment without delayed data (first row) and with delayed sonar measurements recalculating the compass information (third row), making the state and covariances under both setups equal. However, the validation status of the experiment with delayed compass data (second row) and without delayed measurement (first row) is slightly different, because the order of arrival of the information can slightly affect the validation step [15]. This discrepancy justifies the slightly differences that also appear in the *σ_xx_* covariance graphics of the first and second row, because rejecting a piece of information from the sonar S8 visually affects the values of *σ_xx_* as the sensorial information directly associated to the *p_x_* state is really sparse.

The results presented in the fourth row of Figure 6 are significantly different to the ones shown in the previous rows. The discrepancy starts after t = 46 s, where it happens to be a change of the value in *p* in all the scenarios around the indetermination that should be taken into account in order to re-assimilate the non-delayed compass information when the delayed sonar information becomes available. However, as EIFAsyn is not required to recalculate the compass information in the fourth case, the arrival of the delayed sonar information makes the already assimilated compass information be in the wrong side of the indetermination and the estimate of the orientation *p_θ_* incorrect (0.8 rad ≃ 45°). At that moment, EIFAsyn loses track, because the wrong estimated orientation makes it reject the compass and sonar measurements afterwards and wrongly update the robot location and orientation, as well as its covariances, using only the encoder measurements.

When we generate the simulated data 100 times with the same setup, we observe the same type of erroneous results, sooner or later, 46 times. That is, while moving around the indetermination, the compass information requires to be recalculated almost in half of the simulations. The discrepancies associated to the non-recalculation of the sonar information are minimal and negligible in all the tests. Therefore, and in order to avoid the loss of track of the filter when it is embedded in the control architecture of a real robot, we treat the compass and sonar respectively as sensors with strong and soft non-linearities, *i.e.*, only recalculating the compass information.

Finally, the results presented in the fifth row of Figure 6 are different too, because using only the information provide by the encoders, directly related to the linear and angular displacements, makes EIFAsyn increment continuously the covariances of the robot location and orientation (*σ_xx_, σ_yy_, σ_θθ_*) and have a poor estimate of the robot location (*p_x_, p_y_, p_θ_*). Therefore, for this problem it is really important to be able to use the OOS measurements of the compass and sonars efficiently in order to let the robot control software navigate successfully, as the experiment in the following section shows.

#### 3.3.2. Real Experiments

In order to illustrate the behavior of EIFAsyn within the localization module of the control software of a real robot, we place the robot in the same hall with the same known objects and two unknown ones, and let the control software generate the control signals (within the range [0,15] cm/s) based on the robot location estimated by EIFAsyn and the obstacles locations estimated using the occupancy grid and Bayesian filter in [53].

**Figure 7.**
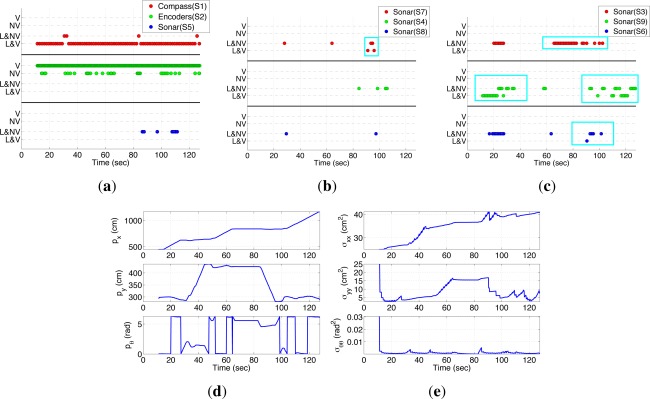
Real Experiments. (**a**) Sensor behavior; (**b**) Sensor behavior; (**c**) Sensor behavior; (**d**) State; (**d**) Covariances.

The results of the experiment are presented in the sensorial behavior, state and covariance graphics of Figure 7. The sensorial behavior graphics (Figure 7(a), 7(b) and 7(c) show that all the measurements, except those provided by the encoders, arrive delayed and out of sequence at the location module. Besides, although the compass and encoder measurements are usually valid, the sonar ones are usually rejected by EIFAsyn, because they are either (1) associated to the unknown objects of the map and can not be used by EIFAsyn or because (2) they are rejected by the EIFAsyn validation test as their error, originated for instance from bounces of the sonar signals in the multiple walls of the bottom U-shape of the hall, is not included in the models. Within the unknown object group fall all the sonar measurements outside the cyan squares in the sensorial behavior graphics, *i.e.*, all the measurements by sonar S5 and S4 (due to the round object), all by S8 (due to the square object), the two first by S7 (due to the square and round object respectively), the first group by S3 (due to the square object), the one at t=60 s by S9 (due to the square object), and the ones by S6 up to t=80 s (due the first group to the square and the second to the round one).

The sparsity of the sonar information makes the OOS filter of the localization module of the control software estimate the robot localization using almost all the compass and encoders information, and only a few measurements provided by the sonars. The small amount of (*p_x_, p_y_*) information makes the covariances of those variables (*σ_xx_, σ_yy_*) grow, while the almost continuous stream of compass measurements makes the angular variance (*σ_θθ_*) really small (see Figure 7(e)). In spite of these facts, EIFAsyn is able to locate properly the robot, as the discrepancy between the real and estimated final robot position falls within the covariance values. Additionally, in this experiment, we can observe how EIFAsyn can need to recalculate the compass information when the measurements of the sonars arrive after the compass ones, due to the abrupt changes of the *p_θ_* values that appear in Figure 7(d) around the angular indetermination. Besides, the control software is able to update the occupancy grid using the robot position estimates and all the sonar measurements. Finally, the reduced computational overload associated to EIFAsyn is negligible inside the control software architecture, which consists of multiple modules of planning and control levels.

### 3.4. Comparison of EIFAsyn with Other General OOS Approaches

In this section we compare, using the robotic problem study in this paper, the behavior of EIFAsyn against the performance and efficiency of some of the general N-step lag OOS algorithms suggested in Section 2.3. The non-linearity in the dynamic model of the robot reduces the possible comparison to the OOS EnKF in [36] and the OOS PFs in [40–42,44,45]. Among all these possibilities, we have decided to analyze the performance of the OOS EnKF in [36] and the OOS PF in [42]-A using the models and simulated data presented in Sections 3.1 and 3.3. With this selection, EIFAsyn is compared against two filtering techniques (EnKF and PF) that differ from the EIFAsyn EKF/EIF support. Besides, our problem tests the OOS EnKF in a more challenging situation that the one presented in [36], because our robot has a non-linear dynamic model with multiple sensors providing information related with different states of the system, and the system in [36] has a linear dynamic model and two equal sensors. Finally, the OOS PF [42]-A (1) equals the OOS PF in [40] when there is a non-delayed measurement (in our case provided by the encoders) for all the measurement time stamps and (2) the selected PF is computationally more efficient than the OOS PFs in [42]-B# and [41,44,45].

In order to carry out the comparison, we have disabled the validation step of EIFAsyn because the other algorithms do not include it and we want all the filters to be able to use the same information. Note that the same validation step can not be included in the other filters because it is not straightforward to obtain in those filters the predicted mean and covariance values used to validate the new measurements in EIFAsyn. Additionally, we compare EIFAsyn with two versions of each of the selected algorithms, because:

- The OOS PF [42]-A estimates the trajectory instead of the state of the system, and therefore, the mean and covariance of the state at any time step *t_i_* are updated with the already assimilated past (*t_k_ < t_i_*), current (*t_k_* = *t_i_*) and future (*t_i_ < t_k_ < t_l_*) measurements ***ξ***_*s,t*_*k*_*,t*_*t*__, while the EIFAsyn mean and covariance only include the already assimilated past and current measurements. In other words, the *standard* OOS PF [42]-A mean and covariance values represent the *p*(***x***_*t*_*i*__*|****ξ***_1:*S,t*_0:*l*__, ***u***_*t*_0_:*t*_*l*__) while the EIFAsyn mean and covariance values represent *p*(***x***_*t*_*i*__*|****ξ***_1:*S,t*_0:*i*__, ***u***_*t*_0_:*t*_*i*__). That is, the standard OOS PF [42]-A mean and covariance values are smoothed with future information that the EIFAsyn values does not use. The *modified* version of OOS PF [42]-A avoids the inherent smoothing behavior of the standard OOS PF [42]-A calculating the mean and covariance values without the future measurements. Therefore, the results obtained by the modified version of OOS PF [42]-A are supposed to be closer to the EIFAsy ones.
- The OOS EnKF [36] runs an EnKF for each sensor and combines the ensembles of each sensor in a unique multisensor fusion ensemble that is used to obtain the mean and covariance of the system. In the problem presented in this paper, this way of proceeding makes the *standard* OOS EnKF [36] run 10 EnKFs (the first for the compass, the second for the encoders, and the remaining eight for all the ultrasonic sensors) and combine the information of many ensembles (in particular those associated to the sonars that are only seeing unrecognizable objects) that have not assimilated any measurement. In other words, as many of the sonar EnKFs are working without measurements, their ensembles contribute only predicted values to the multisensor fusion ensemble. To avoid the influence of these ensembles in the estimated value of the robot localization, the *modified* version of OOS EnKF [36] only combines the results of those ensembles that have updated their state using some measurements.

The two variants of the OOS PF [42]-A and the two variants of OOS EnKF [36] are compared against EIFAsyn using the setup of the simulated experiment and the first, second, third and fifth scenario presented in Section 3.3. The fourth scenario is not considered in this comparison, because it has delayed sonar measurements as the third and it is only required in the EIFAsyn analysis to show that this filter has to recalculate the compass information. Besides, the OOS PFs use 2,000 particles to have often more than 100 effective particles after assimilating each measurement because this number of particles lets us avoid the resampling step after assimilating each measurement, and therefore, it reduces the particles depletion (repeatability of the values of the particles in old time stamps) and increments the effects associated to the weight update produced by delayed measurements. Additionally, the OOS EnKFs use 500 particles in each of the 10 sensor ensembles. That is, we only double the number of particles used in each ensamble in [36], with the hope of solving the performance problems presented in Section 3.4.1, because with that big number of particles the OOS EnKF [36] variants are already the most computationally and memory *inefficient* filters as we will show next. In the following sections we compare the performance, computational load and memory requirements of the 5 filter implementations.

#### 3.4.1. Performance Comparison

**Figure 8.**
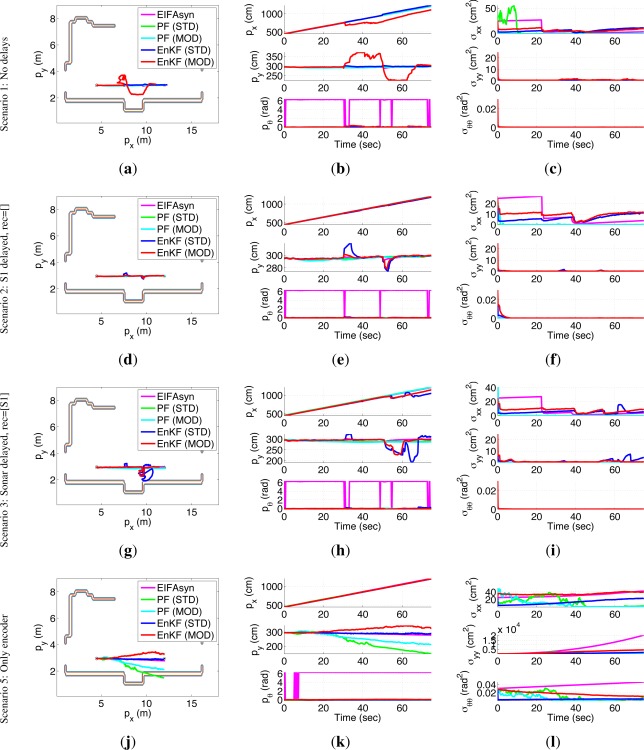
OOS Approaches Comparison.

Figure 8 shows representative results of the 5 filters, using each row for an scenario, each column for a different type of information (the first column represents each filter mean pose, the second the mean values of the robot location, and the third their variances), and a different color for each of the filters (magenta for EIFAsyn, green for the standard version of OOS PF [42]-A, cyan for its modified variant, navy blue for the standard version of OOS EnKF [36], and red for its modified variant).

On one hand, Figure 8(a), 8(b), 8(d), 8(e), 8(g) and 8(h) show the similar values obtained for the estimated locations (the 2*π* variations in *p_θ_* have to be considered negligible too as they occur around the 0–2*π* indetermination) by the PFs variants and EIFAsyn in the first, second, and third scenario, while Figure 8(c), 8(f) and 8(i) show that the PF variants obtain lower variance values than EIFAsyn. Therefore, the three filters obtains similar values for our robot location when the compass and sonar measurements are or are not delayed, with a tighter covariance in the PF cases. As these behavior is observed through different executions of the PFs with the same scenarios and measurements, we can conclude that EIFAsyn and the two PFs variants seem equally valid for our problem.

On the other, Figure 8(a), 8(b), 8(d), 8(e), 8(g) and 8(h) also show how the mean location obtained by any of the EnKF variants sometimes (first and third row) significantly differ from the ones obtained by EIFAsyn (or the PF variants). As a matter of fact, although the EnKF results selected for the second scenario look better than the ones selected for the first and third scenario, the estimated location obtained by other executions of any of the two versions of EnKFs in any of these three scenarios can significantly differ from the ones obtained by EIFAsyn. In other words, none of the EnKF versions works systematically properly with our robotic problem, and the wrong behavior is independent of the scenario delays. A detailed analysis of the estimation process makes us believe that this can be due to the fact that each of the 10 ensembles is corrected independently with measurements that provide information of the different states, and therefore their results can easily diverge due to the lack of the complementary information provided by other sensors to other ensembles. The problem seems to be aggravated by the sonar ensembles that incorporate only sporadic measurements that lack orientation information. It is worth highlighting too that the problem presented in [36] to analyze the behavior of the standard version of OOS EnKF is different to ours because (1) it is characterized by a linear dynamic model without angular information and (2) its two non-linear sensors are equal and provide a periodic stream of delayed measurements. Besides, note that although the results of the standard version of the EnKF seem better in some cases, this can be due to the fact that combining the information of all the ensembles that work without measurements can minimize the divergence effects of the sonar ensembles that are working with sporadic data. Therefore, we can conclude that both versions of EnKF seem invalid for our problem, even in the case when the measurements are not delayed. In spite of this invalidity, we will also compare against others its computational cost and memory requirements to facilitate the selection process of other researchers.

Finally, the performance comparison of the filters in the fifth scenario (fourth row) show that for the robotic problem we need to incorporate the compass and sonar data, because when any of the filters work only with the encoders measurements, the location mean values diverge and the location variances significantly grow.

#### 3.4.2. Computational Comparison

In order to measure the computational load of the 5 filters, we measure for the 4 scenarios presented in the previous section the time required by each of the filters, which are implemented in C++, when they are run over a Intel Core2 Duo at 2.53 GHz with Windows XP. This time is measured in milliseconds with the help of the C++ function GetTickCounts(), which retrieves the number of milliseconds that have elapsed since the PC was started. The resolution of the function, and therefore of our measurements, are in the range of 10 to 18 milliseconds.

The obtained results are presented in Table 3, directly as mean times (columns from 2 to 6) or as ratio time values (columns 7 to 11) with respect the mean time obtained by EIFAsyn for the the same scenario.

The mean time columns show that for each filter, the computational load of the last scenario (where the filters are only predicting and assimilating non-delayed encoder measurements) is smaller than the computational load of the first (where the filters assimilate all the available measurements without delays) and that the first and fifth are lower than the second and third (where the filters assimilate delayed measurements). The similar values within the second and third scenario in all the filters show that each filter is similarly computationally penalized when the delays occur in the compass or in the sonar measurements.

The normalized mean time columns show that for the same scenario EIFAsyn has a significantly lower computational load than the other filters. Besides, the computational load of the modified versions of the selected filters are lower than the computational load of their standard counterparts. In the PF case, the bigger computational load of the standard version is due to the necessity of computing the mean and covariance values of the intermediate states when any delayed measurements arrives. In the EnKF case, the bigger computational load of the standard version is due to the computational difference of fusing the information of 10 or 4 ensembles.

In short, the filters can be sorted according to the computational time, from the best to the worst as: EIFAsyn, PF (MOD), PF (STD), EnKF (MOD), EnKF (STD). Finally, it is worth noting that the linear relationship that exists in the selected PF and EnKF between the number of particles and the computational load of the algorithms will make these PFs/EnKFs with only 200/50 particles (which are are not enough to make any of the filters work properly) still have a computational load one order of magnitude bigger than EIFAsyn. Therefore, the low computational load of the EIFAsyn, in spite of its necessity to recalculate the information associated to the sensors with strong non-linearities, is a plus that can not be underestimated.

#### 3.4.3. Memory Comparison

In order to compare the memory requirements of the 5 filters, we theoretical count the number of floats that are necessary to store the minimal information required to make them assimilate any measurement that is delayed at maximum *W* time steps.

The results of this count are presented in the second column of Table 4, where *N_x_* stands for the number of states of the system, *N_s_* for the number of measurement variables returned by each of the *s* sensors whose information needs to be recalculated, *N_p_* for the number of particles in the algorithm, and *S* for the number of sensors in the system. The EIFAsyn formula is counting, for each time step in *W*, the space associated to 2 state variables + 2 covariance variables + the measurement variables whose information has to to be recalculated. The PFs equation is counting the memory required for saving the total number of particles in the PF in each time step + the weights associated to each PF trajectory. Finally, the EnKF expression is counting the required memory to store the number of particles/elements in each of the S ensembles during the *W* time steps.

The results of the count for our problem (*N_x_* = 5, *N_s_*_=1_ = 1, *S* = 10) and filter configurations (*N_p_* = 2000 for the PFs and *N_p_* = 500 for the EnKFs) are presented in the third column of the table. The worst filter regarding the memory requirements is clearly EnKF, followed closely by the PF. Besides, EIFAsyn memory requirements is two orders of magnitude smaller than the others. Therefore, regarding the memory cost, EIFAsyn is also the best option for the problem.

#### 3.4.4. Overall Comparison

Taking into account the performance comparison, we conclude that while the OOS EnKF variants are invalid for our problem, both the OOS PF variants and EIFAsyn are similarly applicable. Besides, between the two valid possibilities, the memory and computational requirements of EIFAsyn are significantly smaller, making it an ideal choice to be incorporated in the complex control architecture of a robotic system like ours.

## 4. Conclusions

This paper presents a thoughtful analysis of the different possibilities of OOS algorithms that exist for estimating the location of non-linearly modeled robots, taking into account the properties of the existing algorithms and of the robotic problem. Therefore, this analysis can be used to determine which are the best algorithms for different types of non-linearly modeled OOS estimation problems, as well as to know which problems similar to the autonomous mobile robot location estimation one have already been tackled with which algorithms.

Besides, this paper also shows how one of the most generic and efficient OOS algorithms, the EIFAsyn presented in [30,31], can be applied for the localization problem or a real problem. With that purpose, we modify the algorithm slightly in order to take into account some of the particularities of the problem, and we analyze which of the non-linear sensors that provide measurements about the robot position require to have its sensorial information recalculated when the algorithm receives older measurements out-of-sequence. After determining those necessities, we successfully test the behavior of the adapted OOS EIF, making it responsible for estimating the robot location used in all the other algorithms that form part of the complex control software of a real robot.

Finally, this paper also compares the performance, computational cost and memory requirements of EIFAsyn with two of the other techniques that have been selected using the characteristics determined in our thoughtful analysis. The results of the comparison show that the selected algorithm EIFAsyn is the best choice for the localization problem regarding its memory and computational cost and one of the best options regarding its performance.

## Figures and Tables

**Table 1.** OOS Filters for Gaussian Non-Linear Systems.

***ALG***	***Group***	***Extending***	**Support**	***Trans. Model***	***Meas. Model***	***OOSP***	***Extras***
[26]	LKF	[5]-A1	Retrodiction	Lzd	Lzd	1 step-lag	
[27,28]-EB1	EKF	[32]-B1	Retrodiction	L	L/NL	N step-lag	
[29]-EA1	EKF	[33]-Al1	Retrodiction	L	L/NL	N step-lag	
[29]-EB1	EKF	[33]-Bl1	Retrodiction	L	L/NL	N step-lag	
[29]-EFPFD	EKF	[34] & [29]-FPFD	Forward propagation	L	L/NL	N step-lag	
[30,31]-EIFAsyn	EIF	[30,31]-IFAsyn	Forward propagation	L/NL	L/NL	N step-lag	Optional recalculation
[35]	UKF	[22]	Retrodiction	L	L/NL	1 step-lag	
[36]	EnKF	[23]	Linear Interpolation	L/NL	L/NL	N step-lag	
[37]	PF	SIR	$q\left(x_{t_{m}}|x_{t_{a}}^{\left(j\right)},x_{t_{b}}^{\left(j\right)}\right)$	L	L/NL	N step-lag	
[38,39]	PF	SIR	$q\left(x_{t_{m}}|x_{t_{a}}^{\left(j\right)},x_{t_{b}}^{\left(j\right)}\right)$	L	L/NL	N step-lag	MCMC smoothing
[40]	PF	SIR	Linear Interpolation	L/NL	L/NL	N step-lag	
[41]	PF	UPF	$q_{\mathrm{UKF}}\left(x_{t_{m}}|x_{t_{a}}^{\left(j\right)},x_{t_{b}}^{\left(j\right)},z_{s,t_{m}}\right)$	L	L/NL	N step-lag	
[42]-A	PF	SIR	$q_{\mathrm{EKF}}\left(x_{t_{m}}|x_{t_{a}}^{\left(j\right)},x_{t_{b}}^{\left(j\right)}\right)$	L/NL	L/NL	N step-lag	
[42]-B1	PF	MPF	Fixed-point EK Smoother	L/NL	L/NL	N step-lag	Check diversity
[42]-B2	PF	MPF	Fixed-point UK Smoother	L/NL	L/NL	N step-lag	Check diversity
[42]-B3	PF	MPF	Fixed-point Particle Smoother	L/NL	L/NL	N step-lag	Check diversity
[43]	PF	MPF	$q_{\mathrm{Retro}}\left(x_{t_{m}}|x_{t_{k}}^{\left(j\right)}\right)$	L	L/NL	N step-lag	
[44] & [45]	PF	[42]-B#	[42]-B#	L/NL	L/NL	N step-lag	Check diversity

**Table 2.** Constants and Variances of the Models.

**Variable Dimensions**	***dt* (s)**	***b* (cm)**	**Sonar Directivity (rad)**	***var*_Δ*^l^*_ (cm/s)^2^**	***var_Δ^θ^_* (rad/s)^2^**	***var_compass_* (rad)^2^**	***var_N_L__* (cm/s)^2^**	***var_N_R__* (cm/s)^2^**	***var_sonar_* (cm)^2^**
Real Robot Data				(1)^2^	(2*π*/180)^2^	(2*π*/180)^2^	(0.5)^2^	(0.5)^2^	(1.5)^2^
Simulation Data	0.1	24.5	12*π/*180	(1)^2^	(0.25*π/*180)^2^	(2*π/*180)^2^	(0.5)^2^	(0.5)^2^	(1.5)^2^
EIFAsyn Data				(2)^2^	(3*π/*180)^2^	(3*π/*180)^2^	(1)^2^	(1)^2^	(2)^2^

**Table 3.** Computational Cost Comparison.

**Scenarios**	**Mean Measured Time (ms)**					**Normalized Mean Time**				

	**EIFAsyn**	**PF (STD)**	**PF (MOD)**	**EnKF (STD)**	**EnKF (MOD)**	**EIFAsyn**	**PF (STD)**	**PF (MOD)**	**EnKF (STD)**	**EnKF (MOD)**
Scenario 1	1,880	241,010	230,840	602,340	404,860	1	128	123	320	215
Scenario 2	2,010	291,280	283,670	618,840	419,090	1	150	141	307	208
Scenario 3	2,200	294,480	284,940	618,360	415,080	1	134	130	281	189
Scenario 5	320	181,060	170,500	388,950	242,810	1	565	533	1,215	759

**Table 4.** Memory Comparison.

**Filter**	**Theoretical Float Count**	**Applied Float Count (W = 10)**
EIFAsyn	$W\cdot \left(2\cdot (N_{x}+N_{x}^{2})+\sum_{s\in rec}N_{s}\right)$	620
PF (STD & MOD)	*W · N_p_ · N_x_* + *N_p_*	102; 000
EnKF (STD & MOD)	*W · S · N_p_ · N_x_*	250; 000

## Appendix

In the following equations, we show the Jacobians, ***F***_*t,t*−1_ = (***J****f*)*_x_*(***x̂****_t−_*_1_*_|t−_*_1_, ***u****_t,t−_*_1_*, t, t* − 1) and ***H****_s,k_* = (***J****h_s_*)*_x_*(***x̂****_k|k−_*_1_*, k*), of the transition and measurement models. Additionally, the information in Table A1 highlights some relevant aspects that have been considered when implementing the OOS PF and EnKF variants, shortly describing the problem in the first column, determining in which filters has to be considered in the second and third, and introducing the idea behind the solution in the last.

(12) $$F_{t+1,t}=\left(\begin{array}{ccccc} 1 & 0 & -\Delta_{t}^{l}\;\text{sin}(p_{t+1}^{\theta }) & \text{cos}(p_{t+1}^{\theta }) & -\Delta_{t}^{l}\;\text{sin}(p_{t+1}^{\theta })\\ 0 & 1 & \Delta_{t}^{l}\;\text{cos}(p_{t+1}^{\theta }) & \text{sin}(p_{t+1}^{\theta }) & \Delta_{t}^{l}\;\text{cos}(p_{t+1}^{\theta })\\ 0 & 0 & 1 & 0 & 1\\ 0 & 0 & 0 & 0 & 0\\ 0 & 0 & 0 & 0 & 0 \end{array}\right)$$

(13) $$H_{1,t+1}=\left(\begin{array}{ccccc} 0 & 0 & 1 & 0 & 0 \end{array}\right)$$

(14) $$H_{2,t+1}=\left(\begin{array}{ccccc} 0 & 0 & 0 & 1 & -b/2\\ 0 & 0 & 0 & 1 & b/2 \end{array}\right)$$

For *s ∈* [3, 10]:

(15) $$\begin{array}{l} H_{s,t+1}=\{\begin{array}{ll} \left({\mathrm{gA}}_{\mathrm{obj}}\;\;\;\;{\mathrm{gB}}_{\mathrm{obj}}\;\;\;\;g(B_{\mathrm{obj}}\;d_{s}\;\text{cos}(p_{t+1}^{\theta }+\alpha_{s})-A_{\mathrm{obj}}\;d_{s}\;\text{sin}(p_{t+1}^{\theta }+\alpha_{s}))\;\;\;0\;\;\;0\right) & |\;\mathrm{obj}=\mathrm{wall}\\ \left(\frac{p_{t+1}^{x}+d_{s}\;\text{cos}(p_{t+1}^{\theta }+\alpha_{s})-p_{\mathrm{obj}}^{x}}{q}\;\;\;\;\;\frac{p_{t+1}^{y}+d_{s}\;\text{sin}(p_{t+1}^{\theta }+\alpha_{s})-p_{\mathrm{obj}}^{y}}{q}\;\;\;\;w\frac{d_{s}}{q}\;\;\;\;0\;\;\;\;0\right) & |\;\mathrm{obj}=\left\{ \begin{array}{l} \mathrm{corner}\\ \mathrm{column} \end{array}\right. \end{array}\\ \text{with}\;g=\{\begin{array}{ll} 1 & \text{if}|A_{\mathrm{obj}}\;\left[p_{t+1}^{x}+d_{s}\;\text{cos}(p_{t+1}^{\theta }+\alpha_{s})\right]+B_{\mathrm{obj}}\;\left[p_{t+1}^{y}+d_{s}\;\text{sin}(p_{t+1}^{\theta }+\alpha_{s})\right]+C_{\mathrm{obj}}|\;>0\\ -1 & \text{otherwise} \end{array}\\ \;\;\;\;\;\;\;\;\;\;\;q=\sqrt{{\left[p_{t+1}^{x}+d_{s}\;\text{cos}(p_{t+1}^{\theta }+\alpha_{s})-p_{\mathrm{obj}}^{x}\right]}^{2}+{\left[p_{t+1}^{y}+d_{s}\;\text{sin}(p_{t+1}^{\theta }+\alpha_{s})-p_{\mathrm{obj}}^{y}\right]}^{2}}\\ \;\;\;\;\;\;\;\;\;\;\;w=\left[p_{t+1}^{y}-p_{\mathrm{obj}}^{y}\right]\;\;\text{cos}\left(p_{t+1}^{\theta }+\alpha_{s}\right)-\left[p_{t+1}^{x}-p_{\mathrm{obj}}^{x}\right]\;\;\text{sin}\left(p_{t+1}^{\theta }+\alpha_{s}\right) \end{array}$$

**Table A1.** Relevant additional aspects to implement the OOS PFs and EnKFs.

**Problem Description**	**PFs**	**EnKFs**	**Solution**
Calculating mean values of angular variables	x	x	Performing the operation in the cartesian space associated to the polar coordinates of the angle [54]
Resampling step	x		Carrying it out optionally, based on the number of effective particles [55]
The existence of a sonar prediction depends on the state orientation	x		Assigning a 0 weight to those particles from which the object can not be observed, unless no observation can be predicted from any particle
		x	Assimilating the sonar measurement only if an object can be observed from all the particles of the ensemble
